# The prevalence and effective factors of crash helmet usage among motorcyclists in Iran

**DOI:** 10.5249/jivr.v8i1.667

**Published:** 2016-01

**Authors:** Seyed Taghi Heydari, Kamran B. Lankarani, Mehrdad Vossoughi, Kazem Javanmardi, Yaser Sarikhani, Kourosh Mahjoor, Mojtaba Mahmoodi, Mohammad Khabaz Shirazi, Maryam Akbari

**Affiliations:** ^*a*^Health Policy Research Center, School of Medicine, Shiraz University of Medical Sciences, Shiraz, Iran.; ^*b*^Department of Dental Public Health, School of Dentistry, Shiraz University of Medical Sciences, Shiraz, Iran.; ^*c*^Fars Province Police Headquarter Applied Research & Studies Office, Shiraz, Iran.; ^*d*^Research Center for Social Determinants of Health, Jahrom University of Medical Sciences, Jahrom, Iran.

**Keywords:** Crash helmet, Motorcyclist, Risk factors, Iran

## Abstract

**Background:**

Crash helmet plays an important role in protecting the deriver's head during crashes and reduces the rate of severe injuries and fatalities. Although it has been proved that wearing the crash helmet can save the deriver’s life by around 42%; previous studies showed that the rate of wearing crash helmet has not been acceptable in Iran. Due to the huge number of motorcyclists on the roads in Iran, the use of crash helmet is an important area of research. The aim of this study was to assess the factors that could possibly relate to or affect the use of crash helmet by the motorcyclists.

**Methods:**

This is an observational study on 414 motorcyclists in Shiraz, Southern Iran. All participants completed a questioner containing demographic features, crash helmet use, motorcycle license, and the reasons for using motorcycles.

**Results:**

All the participants were males and aged from16 to 64 years with mean age 27±9.28. The results of logistic regression model revealed that only the drivers who had motorcycle license (OR=2.73, C.I: 1.40-7.24), employed the motorcycle for reasons other than pleasure (OR=3.18, C.I: 1.42-7.37) and been driving for 10 or more years (OR=1.92 95% C.I: 1.12-3.30) had greater rate of wearing crash helmet. Interestingly, educational levels, age, and other demographical variables had no relationship with crash helmet usage.

**Conclusions:**

It is believed that in order to increase the rate of crash helmet use, it is necessary to enact obligatory requirement for driving license by motorcyclists and increase the legal age for motorcycle driving.

## Introduction

Iran is a country with the highest rate of traffic accidents involving high mortality and injury.^1-3^ This is evidenced by 22,918 traffic accident deaths recorded in Iran during 2007-2008. ^4^ The major part of mortality and morbidity due to traffic accidents belongs to motorcycle deriver. ^5^ The motorcycle riders are approximately ten times more likely to suffer severe injuries and fatalities than drivers of four-wheeled vehicles. ^6^ A report from World Health Organization (WHO) classified motorcyclists as a group with high risk for injuries. ^7^ Head trauma is the main cause of death in motorcycle accidents. ^8,9^ Safety helmet plays important role in protecting the deriver's head during crashes and reducing the risk of severe injuries and fatalities. After repeal of mandatory helmet wearing laws in some countries, motorcyclist fatalities increased by an average 25-28%.^10-12^ Moreover, the risks of head trauma and death for helmetless riders were 2.30 and 3.19 times more than those who wore crash helmet, respectively.^13^

Although the previous study showed that the helmet wearing can reduce the risk of death and injuries in accidents,^14^ the rate of helmet wearing has not been satisfactory in Iran. For example, two studies conducted in Iran showed that only 11.8% of the motorcyclists reported that they always wore a crash helmet when driving a motorcycle^15^ and about 8-11% of motorcyclists wore crash helmet at the time of their accident. ^16,17^ Also, helmet usage was highly dependent on the time of day and the season.^16^

It is thought that the enforcement and instructional strategies based on behavioral analysis possibly increase the rate of crash helmet usage and decrease the death rate among motorcyclists.^9^ In this context, public media has a crucial role in informing the drivers about the advantages of wearing crash helmet.

In regard to law enforcement, it should be considered that the effect of law on different demographic group may not be similar, an issue deserving careful attention by interventional researches, and in designing preventive and educational programs.

Shiraz is the capital city of Fars, the fifth largest province of Iran, located in Southwest of Iran. According to the report of Statistical Center of Iran, Shiraz had a total population of about 1700000 in 2011.^18^ The mortality rate due to traffic accident in Fars is 42 per 100 000 which is higher than Iran's rate of 31.1 per 100 000. ^18^ The mortality rate of motorcycle drivers was 828 (%22.7) from the total records of 3642 traffic accident deaths in Fars between 2009-2011.^18^ Despite the high rate of mortality in motorcycle accidents, the rate of crash helmet use and its related factors have never been specifically investigated in Iran. The aim of this study was to determine the prevalence and assess the epidemiological aspects of wearing crash helmet by motorcyclists in Shiraz, Iran. 

## Methods

This observational study was conducted in Shiraz, the capital city of Fars province of Iran. 

Data from motorcycle derivers were collected using a questionnaire in particular areas at specific times of day. The questionnaire was designed by some experts from university faculty members, traffic and transportation organization, and the traffic police. The appropriateness of each item in the questionnaire was assessed by them and irrelevant items was removed or modified to improve the validity of the scale. A primary sample of 35 motorcyclists also showed an acceptable level of reliability for the questionnaire (Chronbach’ α = 0.74).

The questionnaire contained demographical variables including age, marital status, living location (city or rural areas), education level, and income level. Also the questionnaire included information about wearing crash helmet in past three months and the reasons for riding motorcycle.

A total of 414 motorcyclists participated in this study. All motorcycle drivers were selected randomly based on their presence in eight major streets or crossings in four geographical areas and at different times of the day. These different places and time intervals were considered with respect to the traffic center of police to provide a representative sample from the motorcyclist population. The goals of study were explained to the drivers who were asked to fill in the questionnaire after giving their written informed consent, in the space provided in the questionnaire, to take part in the study. 

**Statistical Analysis**

Statistical Package for the Social Sciences Version 17.0 (SPSS Inc., Chicago, IL, USA) was used to analyze the data. Frequency (%) and mean ± standard deviation were used as descriptive indices. Chi-square test, odds ratio (OR) and corresponding confidence interval (%95 C.I) were used to assess the univariate relationships between independent variables and wearing crash helmet as dependent variable. 

Throughout our study, the wearing of crash helmet was considered as dependent variable where those who never used or rarely wore crash helmet were also considered as participants. We also estimated the adjusted ORs using a multiple logistic regression as a full model to control the effect of possible confounders, since we were interested in reporting adjusted ORs accompanied by their univariate counterparts which were not provided by the selection methods. The significance level was set at 0.05. 

## Results

A total of 414 motorcyclists participated in the study of whom 246(59.4%) were singles. The participants were males, aged from 16 to 64 years with mean age 27 ± 9.28 years. The wearers of crash helmet defined as users, were 137(33.1%) drivers who always or most often used crash helmet, against 277(66.9%) motorcyclists defined as non-users that never or rarely wore crash helmet. Only 285 (68.8%) derivers held driving license. There were 33(8%) derivers who were under 18 years old, the legal age for getting license. 

Table 1 shows the univariate and adjusted associations of demographic and driving-related variables of wearing crash helmet. The unadjusted results indicated that the drivers in age groups 25-34 (OR =5.37, %95 C.I: 1.80-16.06) and ≥35 (OR=3.97, %95 C.I: 1.24-12.70) had greater rate of using crash helmet compared to those aged lower than 18 years. The married motorcyclists (OR=1.82, %95 C.I: 1.20-2.76), those who used motorcycle for business reasons (OR=3.84, C.I: 1.75-8.33), and those who held license (OR=2.72, C.I: 1.76-4.20) were more likely to use crash helmet.

**Table 1:** Univariate and adjusted associations of demographic and driving-related variables with crash helmet use.

variable		Helmetless	Helmet user	P*	OR† (%95 C.I)	OR‡ (%95 C.I)
	<18	29 (87.9)	4 (12.1)		1	1
Age (year)	18-35	121 (72)	47 (28)	<0.001	2.82 (0.94-8.45)	1.98 (0.63-6.24)
	>35	42 (64.6)	23 (35.4)		3.97 (1.24-12.70)	3.02 (0.90-10.10)
Marital status	Single	178 (72.4)	68 (27.6)	0.004	1	1
	Married	99 (58.9)	69 (41.1)		1.82 (1.20-2.76)	1.43 (0.84-2.42)
	Under diploma	138 (67.6)	66 (32.4)		1	1
Education	Diploma	95 (65.5)	50 (34.5)	0.907	1.10 (0.70-1.73)	1.06 (0.54-2.09)
	University	44 (67.7)	21 (32.3)		1 (0.55-1.81)	1.16 (0.59-2.30)
Living location	Shiraz	257 (68)	121 (32)	0.130	1.70 (0.85-3.39)	1.89 (0.90-3.98)
	Other	20 (55.6)	16 (44.4)		1	1
Salary (× 1000 Rls)	>500	81 (71.7)	32 (28.3)	0.206	1	1
	<500	196 (65.1)	105 (34.9)		1.36 (0.84-2.17)	1.60(0.93-2.72)
Usage times (per week)	<=3	82 (62.6)	49 (37.4)	0.205	1	1
	>3	195 (68.9)	88 (31.1)		0.75 (0.49-1.17)	0.76 (0.47-1.23)
Type of motor	>125 cc	20 (71.4)	8 (28.6)	0.599	1	1
	<125 cc	257 (66.6)	129 (33.4)		1.25 (0.54-2.94)	1.09 (0.41-2.88)
Motorcycle usage reason	Non-pleasure	224 (63.5)	129 (36.5)	<0.001	3.84 (1.75-8.33)	3.18 (1.42-7.37)
	Pleasure	53 (86.9)	8 (13.1)		-	-
License	No	211 (74)	74 (26)	<0.001	1	1
	Yes	66 (51.2)	63 (48.8)		2.72 (1.76-4.20)	2.73 (1.40-7.24)
	<5	67 (72.8)	25 (27.2)		1	1
Usage duration (year)	5-10	82 (61.7)	51 (38.3)	0.205	1.67 (0.94-2.97)	1.62 (0.83-3.15)
	>=10	128 (67.7)	61 (32.3)		1.28 (0.73-2.22)	1.92 (1.12-3.30)

*: Using chi-square test. †: Univariate Odds Ratio (OR) and corresponding %95 confidence interval (C.I). ‡: Adjusted Odds Ratio (OR) and corresponding %95 confidence interval (C.I) computed using a multiple logistic regression model.

The p-values of chi-square tests also confirmed these results. However, the significant association remained after adjustment made for wearing crash helmet only for business purposes (OR = 3.18, C.I: 1.42-7.37) and holding motorcycle license (OR = 2.72 %95 C.I: 1.66-4.46). Additionally, regression analysis showed that motorcyclists with 10 or more years of driving experience (OR = 1.92 95% C.I: 1.12-3.30) were more likely to wear crash helmet. For purpose of adjustment, all variables associated with crash helmet were included in a logistic regression model, considering those who wore and did not wear this protective device, as dependent variable.

## Discussion

It is obvious that motorcycle accidents cannot be completely prevented.^19^ However using proper safety device could decrease deaths and incapacity by head injuries during the motorcycle accidents. ^20^ This study attempted to establish the determining factors associated with wearing crash helmet, a crucial measure to reduce the burden of accidents among motorcyclists. 

Our results revealed the low rate of crash helmet usage among motorcyclists of whom approximately 15.2% reported to wear crash helmet without fail. However, this value was a little higher than 11.8% reported by another survey in Iran.^15^ In a study by Zamani-Alavijeh in Iran,^21^ it was reported that 23% and 10% of motorcyclists used non-standard and standard crash helmet respectively.

In Iran, males are more likely to engage in motorcycle deriving than females; therefore all participants in our study were males. However, studies conducted in other countries showed that women were significantly more likely to wear crash helmet.^22-24^

The results also indicated that adult derivers aged 18-34 and drivers aged 35 years or more used crash helmet more frequently than those under 18 years of age. This is somewhat consistent with the results reported by Hung et al.,^23^ for adult derivers (OR = 8.56). It was also found that the prevalence of wearing crash helmet was significantly higher in married motorcyclists. However, after the adjustment using a logistic regression model the statistical significance was lost. It may due to the uncontrolled effect of age in univariate analysis. Drivers who held motorcycle license were also more likely to wear crash helmet, a finding in line with that of Skalkidou et al., (OR = 1.59). ^22^ The results also showed that using motorcycle for reasons other than pleasure and holding motorcycle license increased the rate of crash helmet use by motorcyclists. 

Kulanthayan et al., in their study in Malaysia demonstrated that compliance with using crash helmet was 69.2% among older people ,65% in female motorcyclists and 58.5% in those with valid license, which were significantly higher than other groups of their study.^19^

After the adjustment using a logistic regression model, factors associated with increased helmet use were holding motorcycle driving license, use of motorcycle for reasons other than pleasure and driving for 10 or more years. Surprisingly, there was no significant association between the education level of derivers and wearing crash helmet. This was in contrast with findings of a study on Malaysian motorcycle drivers indicating that people with higher education level were more likely to use safety helmet.^19^

A 4-year database study in Iran showed a significant decrease in road traffic fatalities and morbidity rates 2 years after implementing safety interventions by traffic police where the death rate decreased from 38.2 per 100,000 in 2004 to 31.8 in 2007 (OR = 0.83, 95% CI = 0.82–0.85). It also showed the crucial role of traffic police interventions on the enforcement of traffic rules.^25^ Another study in Italy demonstrated that the rates of helmet usage and hospital admission among motorcycles drivers were significantly decreased after introduction of the revised crash helmet law.^9^

## Conclusion

Some studies show that usage of crash helmet among Iranian motorcyclist is low. The results of this study indicated that holding motorcycle driving license and using motorcycle for business reasons are positively associated with wearing crash helmet. Mandatory helmet wearing law accompanied by instructional programs could be effective to increase the rate of crash helmet usage.

## References

[1] Heydari ST, Hoseinzadeh A, Ghaffarpasand F, Hedjazi A, Zarenezhad M, Moafian G (2013). Epidemiological characteristics of fatal traffic accidents in Fars province, Iran: a community-based survey. Public Health.

[2] Duperrex O, Bunn F, Roberts I (2002). Safety education of pedestrians for injury prevention: a systematic review of randomised controlled trials. BMJ.

[3] Lankarani KB, Sarikhani Y, Heydari ST, Joulaie H, Maharlouei N, Peimani P (2014). Traffic accidents in Iran, a decade of progress but still challenges ahead. Med J Islam Repub Iran.

[4] Gosselin RA, Spiegel DA, Coughlin R, Zirkle LG (2009). Injuries: the neglected burden in developing countries. Bull World Health Organ.

[5] Heydari ST, Maharlouei N, Foroutan A, Sarikhani Y, Ghaffarpasand F, Hedjazi A (2012). Fatal motorcycle accidents in Fars Province, Iran: a community-based survey. Chin J Traumatol.

[6] Li Y, Qiu J, Liu GD, Zhou JH, Zhang L, Wang ZG (2008). Motorcycle accidents in China. Chin J Traumatol.

[7] Zamani-Alavijeh F, Niknami S, Mohammadi E, Montazeri A, Ghofranipour F, Ahmadi F (2009). Motorcyclists' reactions to safety helmet law: a qualitative study. BMC Public Health.

[8] Mayrose J (2008). The effects of a mandatory motorcycle helmet law on helmet use and injury patterns among motorcyclist fatalities. J Safety Res.

[9] Servadei F, Begliomini C, Gardini E, Giustini M, Taggi F, Kraus J (2003). Effect of Italy's motorcycle helmet law on traumatic brain injuries. Inj Prev.

[10] Dee TS (2009). Motorcycle helmets and traffic safety. J Health Econ.

[11] Chenier TC, Evans L (1987). Motorcyclist fatalities and the repeal of mandatory helmet wearing laws. Accid Anal Prev.

[12] Evans L, Frick MC (1988). Helmet effectiveness in preventing motorcycle driver and passenger fatalities. Accid Anal Prev.

[13] Heilman DR, Weisbuch JB, Blair RW, Graf LL (1982). Motorcycle-related trauma and helmet usage in North Dakota. Ann Emerg Med.

[14] Mohan D (2002). Road safety in less-motorized environments: future concerns. Int J Epidemiol.

[15] Aghamolaei T, Tavafian SS, Madani A (2011). Prediction of helmet use among Iranian motorcycle drivers: an application of the health belief model and the theory of planned behavior. Traffic Inj Prev.

[16] Zargar M, Khaji A, Karbakhsh M (2006). Pattern of motorcycle-related injuries in Tehran, 1999 to 2000: a study in 6 hospitals. East Mediterr Health J.

[17] Ali M, Saeed MM, Ali MM, Haidar N (2011). Determinants of helmet use behaviour among employed motorcycle riders in Yazd, Iran based on theory of planned behaviour. Injury.

[18] Heydari ST, Hoseinzadeh A, Sarikhani Y, Hedjazi A, Zarenezhad M, Moafian G (2013). Time analysis of fatal traffic accidents in Fars Province of Iran. Chin J Traumatol.

[19] Kulanthayan S, Umar RS, Hariza HA, Nasir MT, Harwant S (2000). Compliance of proper safety helmet usage in motorcyclists. Med J Malaysia.

[20] Dennis J, Ramsay T, Turgeon AF, Zarychanski R (2013). Helmet legislation and admissions to hospital for cycling related head injuries in Canadian provinces and territories: interrupted time series analysis. BMJ.

[21] Zamani-Alavijeh F, Bazargan M, Shafiei A, Bazargan-Hejazi S (2011). The frequency and predictors of helmet use among Iranian motorcyclists: A quantitative and qualitative study. Accid Anal Prev.

[22] Skalkidou A, Petridou E, Papadopoulos FC, Dessypris N, Trichopoulos D (1999). Factors affecting motorcycle helmet use in the population of Greater Athens, Greece. Inj Prev.

[23] Hung DV, Stevenson MR, Ivers RQ (2006). Prevalence of helmet use among motorcycle riders in Vietnam. Inj Prev.

[24] Xuequn Y, Ke L, Ivers R, Du W, Senserrick T (2011). Prevalence rates of helmet use among motorcycle riders in a developed region in China. Accid Anal Prev.

[25] Soori H, Royanian M, Zali AR, Movahedinejad A (2009). Road traffic injuries in Iran: the role of interventions implemented by traffic police. Traffic Inj Prev.

